# What link between violence against women and self-esteem?

**DOI:** 10.1192/j.eurpsy.2024.1676

**Published:** 2024-08-27

**Authors:** M. Abdelkefi, R. Feki, A. Turki, I. Gassara, N. Smaoui, S. Omri, N. Charfi, L. Zouari, J. Ben Thabet, M. Maalej Bouali, M. Maalej

**Affiliations:** Psychiatry C department, Hedi Chaker university hospital, sfax, Tunisia

## Abstract

**Introduction:**

Violence against women is a public health problem worldwide and a violation of human rights. It affects women’s lives due to its potential short-, medium- or long-term physical and psychological consequences.

**Objectives:**

The aim of our study is to explore the link between violence against women and self-esteem.

**Methods:**

A descriptive cross-sectional study was conducted from March to August 2023 among Tunisian women consulting in three health care centers in Sfax, Tunisia. We have included women victims of violence (psychological, physical, sexual, and economic). We have used a semi-structured interview and the Rosenberg scale to determine the quality of self-esteem.

**Results:**

Among one hundred interviewed women, fifty-four women who had reported being violence victims were included in our study. The mean age of the participants was 44 years with the majority being married (87 %). Only 29.6% had a high school level and 51.9% had a profession. A total of 29.6% had a low socioeconomic status.

We found that 79.6% are victims of domestic violence (57.4% being victims of spousal violence). Psychological violence seemed to be the most frequent type (59.3%).

Almost all those who were abused (90.6%) experienced psychological (emotional) violence.

The mean score of the Rosenberg self-esteem scale was 31.54.

Self-esteem was very low in 16.7%, low in 37%, medium in 18.5%, and high in 27.8% of the women.

A statistically significant association was found between being a victim of spousal violence and low self-esteem (p=0.032). The semi-structured interview demonstrates that women with low self-esteem are more likely to accept violence.

**Conclusions:**

These results justify the implementation of screening and support programs for women victims of violence to improve their self-esteem.

**Disclosure of Interest:**

None Declared

